# Global health in the age of AI: Safeguarding humanity through collaboration and action

**DOI:** 10.1371/journal.pgph.0002778

**Published:** 2024-01-11

**Authors:** Carlos A. Faerron Guzmán

**Affiliations:** Graduate School, University of Maryland, Baltimore, Maryland, United States of America; PLOS: Public Library of Science, UNITED STATES

## Abstract

This opinion article discusses the impact of artificial intelligence (AI) on global health, addressing its potential risks and benefits to the field. It suggests that, given the existential risks of AI development, the global health community must contribute to AI-related advances, ensuring health equity and the wellbeing of vulnerable populations. Through transdisciplinary collaborations, robust AI governance, and an emphasis on equity, strategies are proposed to harness the potential of AI to reduce health inequalities and improve wellbeing at global and local levels.

Artificial intelligence (AI) technologies are rapidly transforming our world and bring with them considerable potential to enhance global health and wellbeing [1]. The influence of AI in healthcare is transformative and can be positive, with capabilities such as diagnosing conditions undetectable to humans and designing innovative, lifesaving pharmaceuticals [2]. These developments augur a future where healthcare systems globally could see significant improvements in health outcomes. AI could even revolutionize our approach to disease prevention and management, ultimately saving countless lives and resources.

While AI offers undeniable potential benefits to wellbeing, experts are also concerned about potential threats. In a recent Stanford poll, a third of AI experts suggested that misuse of AI could result in a “nuclear-like catastrophe”, which could directly impact global health systems and compromise patient safety and healthcare access [3]. An open letter from leading tech experts further highlights the pressing need to address potential health threats from AI, such as exacerbated health inequities and unpredictable public health crises [4]. Examples of this can range from AI-driven medical algorithms that ill-diagnose diseases to AI-generated biotechnology that unintentionally or intentionally creates or modifies life-threatening pathogens. These risks, mainly resulting from unintentional, unprogrammed, and unpredictable AI capabilities, present unique challenges for AI and global health communities [5]. The paradox of AI’s potential as a path for health improvement and as a multiplier of health threats emphasizes the need for a balanced approach to AI implementation and governance.

Careful navigation is required to maximize benefits and minimize harms in this transformative landscape. As we stand at this busy crossroads, with clashing global health challenges creating syndemics and emerging threats, we face a critical choice: we can either harness the power of AI to reduce health inequities or allow it to exacerbate them.

AI’s potential to negatively impact human wellbeing poses several risks to global health at different levels and scales, with diverse implications for countries in different stages of their development. For example, it is likely that for-profit models of AI companies will translate into the deployment of beneficial technologies to human health, mainly to people in high-income countries first. Additionally, minorities and people living in low-income countries, will be impacted by AI systems that rely on data that might be biased and may sustain historical inequities and exclusions in healthcare policies, guidelines, and predictive models. The algorithmic-led denial of insurance coverage that discriminates against marginalized groups, such as people belonging to certain racial or ethnic minorities, is one example of this. The little transparency and lack of accountability mechanisms around some AI technology also threaten data confidentiality and privacy issues [6]. Additionally, disinformation campaigns designed and fueled by AI can be a threat to public health messaging. AI algorithms can potentiate, generate and/or disseminate false information about, for example, vaccine safety and spread it through social media platforms, undermining public health [7]. Most drastically, AI that is not aligned with the goals of humans has the potential to disrupt the very systems that underpin our wellbeing (e.g., information systems, supply-chain systems, economic systems, essential utilities) and those that threaten our very existence (e.g., nuclear weapons).

Because of these diverse levels of impact, mitigating AI’s threats to health necessitates global health professionals’ involvement at every stage of the AI lifecycle. These include clinicians, public health experts, policymakers, civil society, and bioethicists. They should engage in AI’s conceptualization and design, ensuring technologies prioritize the wellbeing of all communities, particularly the vulnerable. Moreover, their active participation in creating accountability structures for AI’s use in healthcare is essential. The global health community can advocate for ethical and equitable AI policies, collaborate in integrating AI systems within existing health infrastructure, and contribute to guidelines to control AI’s public health implications. Essentially, they must use their expertise to guide AI’s evolution towards health equity and global benefit. We have seen this approach in action in the global health community’s response to environmental challenges like climate change–an existential threat that demands actions from every sector of society [8]. These collaborative responses, involving advocacy, policy development, public communication strategies, and coordinated action, offer valuable lessons for informing our approach to tackling AI-related challenges.

From these cooperative approaches, a fundamental component that must emerge is robust mechanisms for AI governance and regulation, such as a Global AI Treaty (e.g., similar to the Pandemic Prevention, Preparedness, and Response Accord currently being negotiated). A potential Global AI Treaty should establish accountability mechanisms and international regulations to ensure AI, globally (i.e., in every nation-state), is designed and deployed safely, ethically, and equitably. Ideally, it would seek to prohibit the most deleterious uses of AI, like autonomous weapons, to regulate the more nuanced uses, such as mass surveillance. A Global AI Treaty would also seek to create norms around transparency and algorithmic fairness to mitigate risks from AI systems that are biased, lack transparency, or compromise confidentiality. Notably, the treaty would provide a framework for holding nations, private companies, and other actors accountable for upholding ethical (and mutually agreed upon) AI principles. If designed inclusively through multi-stakeholder dialogues, including the voices of the global health community, a Global AI Treaty could balance the line between precaution and progress.

The failure to do so effectively and swiftly might put us in a race to weaponize AI or have AI escape our control (i.e., misalign with our own goals) with potentially catastrophic consequences [9]. Furthermore, lack of global coordination will once again leave countries with few tools and poorly equipped to address the rapidly surging health challenges, potentially leading to dire consequences for global health (similar to what happened early on during the HIV epidemic when HIV devastated countries that could not access medications due to a failure of equitable global health governance). Global health professionals have immense experience in successful global treaties (e.g., the WHO Framework Convention on Tobacco Control) to protect our wellbeing. These experiences, both successful and unsuccessful, are invaluable assets in quickly navigating the need for a comprehensive AI framework for international cooperation and regulation.

Looking ahead, the global health community’s expertise in equity and community-centered approaches can help guide an ethical AI future. We can learn from the experience of activists who urged an ethical approach to COVID-19 vaccine distribution and ensure that ethics are at the center of all AI-related treaties and policies. Keeping equity in mind will help us guide where to best build infrastructure, distribute drugs and medical supplies, where to invest in capacity building, and where education is urgently needed. It will also allow us to cater and design with the needs of specific populations in mind, especially those that are the most vulnerable. Community engagement will also be central to developing and implementing AI technologies, ensuring that diverse voices and needs are heard and met, and ensure AI solutions that are more relevant, effective, and culturally sensitive.

Whether we realize it or not, the age of AI is upon us, and its impact on global health cannot be overlooked. In the first months of 2023, as large language models (the technological framework that gave us GPT-4) took the world by storm, a global health colleague told me not to worry, that all the technology did was "predict the next word in a sentence." I ask you, the reader, not to take the potential capacities of AI models lightly. The tech industry and leaders from within have started to raise their concerns (8). Urgency is paramount as we are already on track for a disorganized and inequitable rollout of AI technology, with increasingly hard-to-predict impacts. As we move into an AI-driven future, global health professionals must actively shape AI development and policies, foster transdisciplinary collaboration, and address AI-driven health inequities. The lessons learned by the very nature of our field, in addition to the equitable framework that serves as a common value to the global health community, locate us at a vantage point that we must not take for granted. Let us create a path that harnesses the power of AI to reduce, rather than increase, the gaps that exist in health and wellbeing, for the benefit of all humanity.

## References

[1] Ord T. The precipice: Existential risk and the future of humanity. Hachette Books; 2020.

[2] Busnatu Ș., Niculescu A., Bolocan A., Petrescu G., Păduraru D., Năstasă I., et al. Clinical Applications of Artificial Intelligence—An Updated Overview. J Clin Med. 2022;11(8): 2265. doi: 10.3390/jcm11082265 35456357 PMC9031863

[3] Stanford University Human-Centered Artificial Intelligence. AI Index 2023. 2023 [cited 2023 Nov 21]. In: Stanford University Human-Centered Artificial Intelligence [Internet]. Stanford, California. https://hai.stanford.edu/research/ai-index-2023

[4] Future of Life Institute. Pause giant AI experiments: An open letter. 2023 March 22 [cited 2023 Nov 21]. In: Future of Life Institute [Internet]. https://futureoflife.org/open-letter/pause-giant-ai-experiments

[5] Hogart I. We must slow down the race to God-like AI. 2023 April 12 [cited 2023 Nov 21]. In: Finantial Times [Internet]. https://www.ft.com/content/03895dc4-a3b7-481e-95cc-336a524f2ac2

[6] Lu S. Data Privacy, Human Rights, and Algorithmic Opacity. Cal. L. Rev. 2022;110: 2087.

[7] Leslie D., Mazumder A., Peppin A,. Wolters M., Hagerty A. Does "AI" stand for augmenting inequality in the era of covid-19 healthcare? BMJ. 2021;372: n304. doi: 10.1136/bmj.n304 33722847 PMC7958301

[8] Kreslake J., Sarfaty M., Roser-Renouf C., Leiserowitz A., Maibach E. The critical roles of health professionals in climate change prevention and preparedness. Am J Public Health. 2018 Apr;108(S2):S68–9. doi: 10.2105/AJPH.2017.304044 29072941 PMC5922192

[9] Harris, T., Raskin, A. The AI Dilemma. Center for Humane Technology [Podcast]; 2023. https://www.humanetech.com/podcast/the-ai-dilemma

